# Very-low-density lipoprotein triglyceride and free fatty acid plasma kinetics in women with high or low brown adipose tissue volume and overweight/obesity

**DOI:** 10.1016/j.xcrm.2023.101370

**Published:** 2024-01-16

**Authors:** Maria Chondronikola, Jun Yoshino, Raja Ramaswamy, Joseph Daniel Giardina, Richard Laforest, Richard L. Wahl, Bruce W. Patterson, Bettina Mittendorfer, Samuel Klein

**Affiliations:** 1 Center for Human Nutrition, Washington University School of Medicine, St. Louis, MO, USA; 2 Wellcome-MRC Institute of Metabolic Science-Metabolic Research Laboratories, Medical Research Council Metabolic Diseases Unit, University of Cambridge, Cambridge, UK; 3 Department of Nutritional Sciences and Dietetics, Harokopio University of Athens, Kallithea, Greece; 4 Department of Radiology, Washington University School of Medicine, St. Louis, MO, USA; 5 Sansum Diabetes Research Institute, Santa Barbara, CA, USA

**Keywords:** brown fat, lipids, free fatty acids, triglycerides, obesity, metabolism, kinetics, women

## Abstract

Although a high amount of brown adipose tissue (BAT) is associated with low plasma triglyceride concentration, the mechanism responsible for this relationship in people is not clear. Here, we evaluate the interrelationships among BAT, very-low-density lipoprotein triglyceride (VLDL-TG), and free fatty acid (FFA) plasma kinetics during thermoneutrality in women with overweight/obesity who had a low (<20 mL) or high (≥20 mL) volume of cold-activated BAT (assessed by using positron emission tomography in conjunction with 2-deoxy-2-[^18^F]-fluoro-glucose). We find that plasma TG and FFA concentrations are lower and VLDL-TG and FFA plasma clearance rates are faster in women with high BAT than low BAT volume, whereas VLDL-TG and FFA appearance rates in plasma are not different between the two groups. These findings demonstrate that women with high BAT volume have lower plasma TG and FFA concentrations than women with low BAT volumes because of increased VLDL-TG and FFA clearance rates. This study was registered at ClinicalTrials.gov (NCT02786251).

## Highlights

• Plasma TG and FFA concentrations are lower in women with high BAT than low BAT volume
• Women with high BAT volume have lower plasma TG due to faster VLDL-TG plasma clearance rate
• Women with high BAT volume have lower FFA due to faster FFA plasma clearance rate
• BAT volume is an independent predictor of plasma FFA clearance rate

## Graphical abstract

**Figure:**
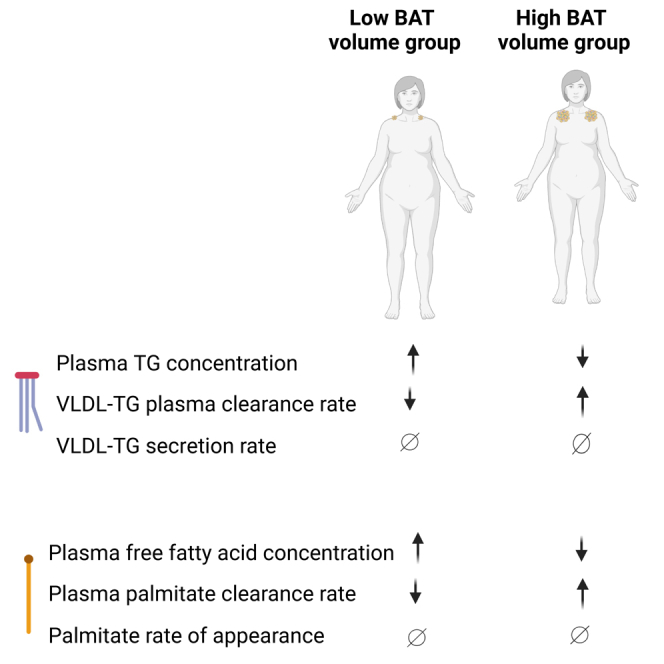

## Teaser

Chondronikola et al. report that women with high brown adipose tissue (BAT) volume have lower plasma triglycerides and free fatty acid concentrations than women with low BAT because of increased very-low-density lipoprotein triglyceride (VLDL-TG) and FFA clearance rates.

## Introduction

High plasma triglyceride (TG) concentration is an important risk factor for coronary heart disease.^1^^,^^2^ In the postabsorptive state, very-low-density lipoprotein (VLDL) particles produced by the liver are the major carrier of circulating TG.^3^ Each VLDL particle contains one apolipoprotein B-100 (apoB-100) “scaffolding” molecule and several thousand TG molecules, plus free and esterified cholesterol, phospholipids, and other apolipoproteins.^3^ Fatty acids released from subcutaneous adipose tissue TG lipolysis are the major source of circulating free fatty acids (FFAs) and fatty acids for VLDL-TG synthesis during basal conditions.^4^^,^^5^^,^^6^ An increase in plasma FFA concentration and FFA delivery to the liver can stimulate hepatic TG production and secretion of TG-rich VLDL particles.^7^^,^^8^ VLDL particles secreted by the liver undergo lipoprotein-lipase-mediated delipidation in extrahepatic tissues (i.e., skeletal and cardiac muscles and adipose tissue), which take up TG-derived FFAs to use as an energy source or for storage, and remnant particles are subsequently cleared by the liver.^9^ Data from studies in mice demonstrate that cold-activated brown adipose tissue (BAT) can decrease plasma lipid concentrations because of its high capacity for TG and FFA uptake and oxidation.^10^^,^^11^^,^^12^^,^^13^ In addition, several studies in people found that a high BAT volume, defined as adipose tissue depots with high glucose uptake, presumably because of a high number of brown adipocytes, is associated with lower plasma TG concentration.^14^^,^^15^^,^^16^ However, the metabolic mechanisms responsible for the difference in plasma TG concentrations between people with high and low amounts of BAT are not known.

The purpose of the present study was to test the hypothesis that people with a large amount of BAT have low plasma TG and FFA concentrations because of increased VLDL-TG and FFA clearance rates rather than decreased secretion rates into the circulation. Stable isotopically labeled palmitate, glycerol, and leucine tracers and mathematical modeling were used to evaluate the relationships among BAT, VLDL-TG, VLDL-apoB, and FFA kinetics and concentrations in women with overweight or obesity, who had either a low amount of BAT (low BAT [LBAT] group, BAT volume < 20 mL) or a high amount of BAT (high BAT [HBAT] group, BAT volume ≥ 20 mL). Positron emission tomography-computed tomography (PET-CT) of the neck and torso after intravenous 2-deoxy-2-[^18^F]-fluoro-glucose ([^18^F]FDG) injection and exposure to mild cold was used to assess BAT volume.

## Results

### BAT volume and metabolic characteristics of the participants

**Figure 1:**
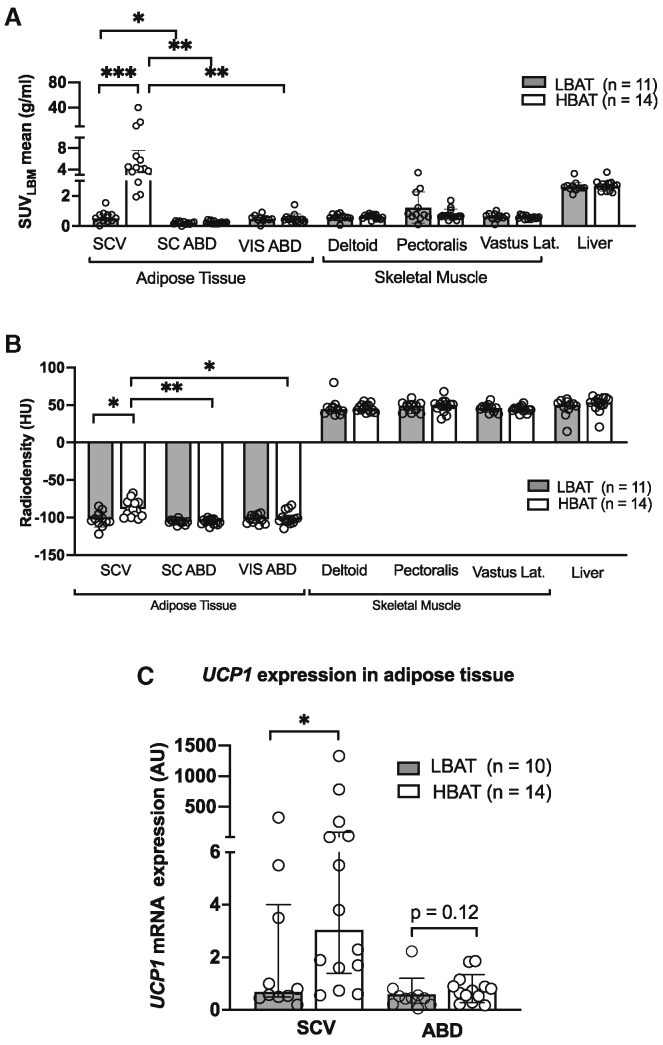
Tissue-specific metabolic assessment (A and B) Mean SUV_mean_ for glucose, assessed by using 2-deoxy-2-[^18^F]-fluoro-glucose positron emission tomography-computed tomography and normalized to lean body mass determined by using dual X-ray absorptiometry (A) and radiodensity assessed using computed tomography (B) of different tissues. p values for within-group comparisons were determined by using paired t test (normally distributed data) or the Wilcoxon rank-sum tests (skewed data). p values for between-group comparisons were determined by using Student’s t test (normally distributed data) or the Mann-Whitney U test (skewed data). Bonferroni correction has been used to correct the reported p values for multiple comparisons. (C) Expression of *UCP1* mRNA assessed by using reverse-transcription polymerase chain reaction in supraclavicular and abdominal adipose tissue samples from the LBAT (n = 10) and HBAT (n = 14) groups. p values were determined by using the Mann-Whitney U test. Data are means ± SD for normally distributed data or median (interquartile range) for skewed data. ABD, abdominal; BAT, brown adipose tissue; HBAT, high-BAT group, BAT volume ≥20 mL; LBAT, low-BAT group, BAT volume <20 mL; HU, Hounsfield units; LBM, lean body mass; SC, subcutaneous; SCV, supraclavicular; SUV, standardized uptake value; UCP1, uncoupling protein 1; VIS, visceral. ∗p < 0.05, ∗∗p < 0.01, and ∗∗∗p < 0.001. Related to Figures S1 and S4 and Tables S1–S3.

Twenty-five women (11 in the LBAT group; 14 in the HBAT group) completed all study procedures related to the primary study outcome (VLDL-TG kinetics) (Figure S1). The HBAT group was about 12 years younger than the LBAT group, but there were no differences in total body adiposity, menopausal status, or markers of glucose control (including fasting plasma glucose concentration, oral glucose tolerance, and insulin sensitivity) between the two groups (Table 1). Energy and macronutrient intakes were not different between the two groups (Table S1). Fasting plasma TG, FFA, and VLDL-apoB concentrations were significantly lower and VLDL-TG concentration was 44%, but not statistically significantly (p = 0.13), lower in the HBAT group than in the LBAT group (Table 1). Adipose tissue metabolic activity ([^18^F]FDG uptake assessed by using PET-CT imaging) and total BAT volume (adipose tissue volume with high metabolic activity) in the neck and torso after 6 h of mild cold exposure were more than 28-fold higher in the HBAT than the LBAT group (Table 1). In addition, [^18^F]FDG uptake was higher in supraclavicular adipose tissue than in both subcutaneous abdominal and visceral adipose tissue depots in the HBAT, but not the LBAT, group (Figure 1A). Tissue radiodensity, which is negatively correlated with tissue lipid content and directly correlated with tissue perfusion,^17^ was higher in the supraclavicular region of participants in the HBAT than the LBAT group and higher in the supraclavicular region than in abdominal subcutaneous and visceral adipose tissue in the HBAT, but not the LBAT, group (Figure 1B). Gene expression of supraclavicular and subcutaneous abdominal adipose tissue samples was consistent with the data obtained by PET-CT imaging and demonstrated about a 4-fold higher expression of *uncoupling protein 1* (*UCP1*), the primary signature protein of BAT,^18^ in supraclavicular adipose tissue in the HBAT compared to the LBAT group (Figure 1C). There was no significant difference in subcutaneous adipose tissue *UCP1* expression between the HBAT and LBAT groups (Figure 1C).

**Table 1:** Participants’ characteristics

	LBAT (n = 11)	HBAT (n = 14)
Age (years)	48.0 ± 7.7	36.0 ± 9.1^a^
Race (White/Black)	10/1	11/3
Menopausal status (pre/post)	8/3	12/2
BMI (kg/m^2^)	29.4 ± 2.3	31.1 ± 2.1
Body weight (kg)	79.7 ± 8.0	86.7 ± 9.9
Height (cm)	165 ± 8.11	167 ± 6.4
Fat-free mass (kg)	46.1 ± 5.6	47.8 ± 4.3
Body fat (%)	42 ± 5	45 ± 3
Subcutaneous abdominal adipose tissue mass (g)	1,933 ± 505	2,323 ± 444
Visceral adipose tissue mass (g)	848 ± 444	939 ± 464
Subcutaneous:visceral abdominal adipose tissue mass ratio	2.9 ± 1.7	3.2 ± 2.3
Fasting plasma glucose (mg/dL)	88.5 ± 8.4	89.8 ± 5.5
Fasting plasma insulin (mU/L)	9.7 ± 5.3	10.1 ± 5.0
HOMA-IR	2.2 ± 1.3	2.3 ± 1.2
2 h OGTT glucose (mg/dL)^b^	124 (117, 152)	122 (111, 130)
Total free fatty acid (μmol/mL)	0.66 ± 0.19	0.47 ± 0.10^c^
Palmitate (μmol/mL)	0.15 ± 0.03	0.12 ± 0.03^c^
Total TG (mg/dL)	97.3 (85.2, 127.4)	70.9 (51.7, 119.1)^c^
VLDL-TG (mg/dL)	53.3 (35.1, 61.6)	29.8 (21.0, 54.8)
VLDL-apoB (mg/dL)	5.6 ± 2.2	3.6 ± 2.1^c^
HDL-cholesterol (mg/dL)	56.1 ± 14.8	54.9 ± 11.4
BAT_LBM_ volume (mL)	2.6 (1.6, 3.6)	72.9 (47.3, 132.0)^d^
Total BAT_LBM_ activity (mL × SUV_mean_)	5.5 (4.7, 9.4)	256.0 (150.5, 562.0)^d^
Total BAT SUV_LBM__mean_ (g/mL)	2.4 (2.3, 2.7)	3.5 (3.3, 3.8)^d^
Total BAT SUV_LBM max_ (g/mL)	4.0 (3.0, 4.8)	12.7 (8.8, 17.6)^d^
Total BAT_LBM_ radiodensity (HU)	−70.7 ± 14.5	−75.2 ± 8.2

Data are means ± SD for normally distributed data or median (quartiles) for skewed data. p values were determined by using the Student’s t test (normally distributed data) or the Mann-Whitney U test (skewed data). ApoB, apolipoprotein B; BAT, brown adipose tissue; HBAT, high-BAT group, BAT volume ≥20 mL; LBAT, low-BAT group, BAT volume <20 mL; BMI, body mass index; HOMA-IR, homeostasis model assessment of insulin resistance; HUs, Hounsfield units; LBM, lean body mass; OGTT, oral glucose tolerance test; SUV, standardized uptake value; SUV_LBM_, SUV normalized to LBM; TGs, triglycerides; VLDL, very-low-density lipoprotein. Related to Figures S1 and S4.

a p < 0.01.

b Data missing for one participant in the LBAT group.

c p < 0.05.

d p < 0.001.

### Plasma FFA and VLDL kinetics

**Figure 2:**
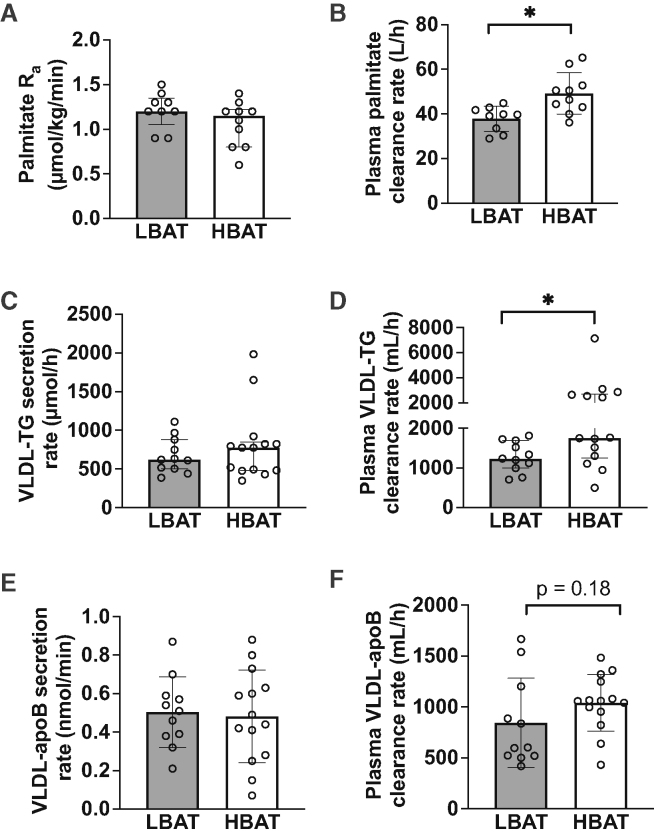
Plasma palmitate, VLDL-TG, and VLD-apoB kinetics in women with low and high amounts of detectable BAT (A and B) Plasma palmitate R_a_ (A) and plasma clearance rate (B) in the LBAT (n = 9) and HBAT (n = 10) groups. (C–F) VLDL-TG secretion rate (C), plasma clearance rate (D), VLDL-apoB secretion rate (E), and plasma clearance rate (F) in the LBAT (n = 11) and HBAT (n = 14) groups. VLDL-TG and VLDL-apoB kinetics were assessed by using intravenous infusion of stable isotopically labeled glycerol and leucine tracers and compartmental modeling analysis. A smaller number of participants completed assessment of fatty acid kinetics, as this assessment was added later in the study protocol. ApoB, apolipoprotein B; BAT, brown adipose tissue; HBAT, high-BAT group, BAT volume ≥20 mL; LBAT, low-BAT group, BAT volume <20 mL; R_a_, rate of appearance; VLDL-TG, very-low-density lipoprotein triglycerides. Data are means ± SD for normally distributed data or median (interquartile range) for skewed data. p values for between-group comparisons were determined by using Student’s t test (normally distributed data) or the Mann-Whitney U test (skewed data). ∗p < 0.05. Related to Figures S2 and S5.

Palmitate rates of appearance (R_a_) in plasma, which equal body palmitate disposal rate from plasma (i.e., molar amount of plasma palmitate removed per minute) during steady-state conditions after subjects fasted overnight, were not different between the HBAT and LBAT groups (Figure 2A). However, plasma palmitate clearance rate (i.e., the volume of plasma cleared of palmitate per hour) was higher in the HBAT than in the LBAT group (Figure 2B).

VLDL-TG secretion rates, which equal VLDL-TG disposal rate during steady-state conditions, were not different between the HBAT and LBAT groups (Figure 2C). However, VLDL-TG plasma clearance rate (the volume of plasma cleared of VLDL-TG per hour) was higher in the HBAT than the LBAT group (Figure 2D). The contributions of systemic FFA (derived primarily from lipolysis of subcutaneous adipose tissue TG) and non-systemic fatty acids (derived from lipolysis of intra-abdominal, intrahepatic, and circulating lipids and *de novo* hepatic lipogenesis) to VLDL-TG production were not different between the HBAT and LBAT groups (Figure S2). VLDL-apoB secretion rates were not different between groups (Figure 2E), and although the VLDL-apoB clearance rate was about 20% faster in the HBAT than the LBAT group, the difference was not statistically significant (p = 0.18) (Figure 2F).

**Figure 3:**
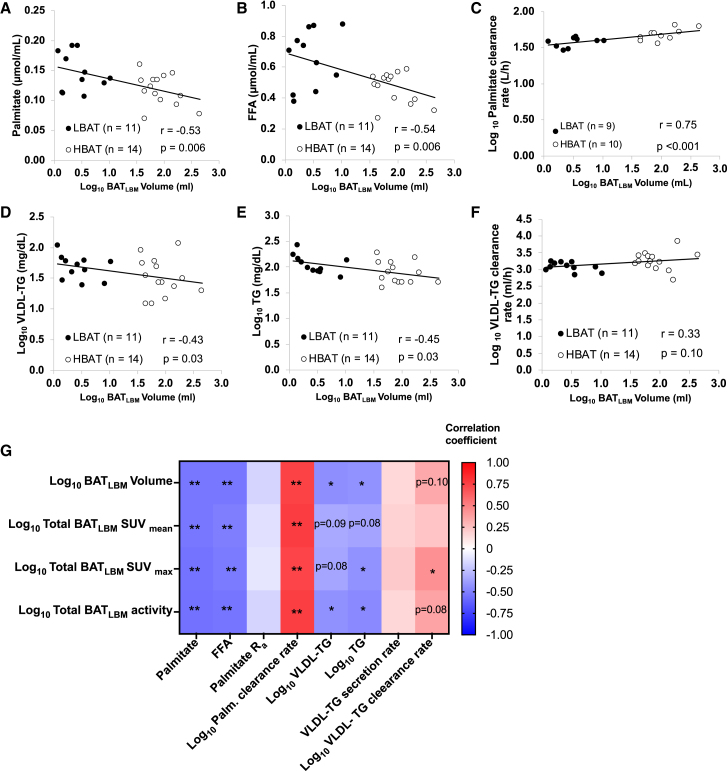
Relationships among BAT, lipid concentrations, kinetics (A) Relationship between BAT volume and plasma palmitate concentration. (B) Relationship between BAT volume and plasma FFA concentration. (C) Relationship between BAT volume and palmitate clearance rate. (D) Relationship between BAT volume and VLDL-TG concentration. (E) Relationship between BAT volume and plasma TG concentration. (F) Relationship between BAT volume and VLTG clearance rate. (G) Heatmap depicting the interrelationships between BAT volume and activity and lipid concentrations and kinetics. BAT, brown adipose tissue; BAT_LBM_, BAT normalized to lean body mass; HBAT, high-BAT group, BAT volume ≥20 mL; LBAT, low-BAT group, BAT volume <20 mL; FFA, free fatty acids, R_a_, rate of appearance; SUV, standardized uptake value; VLDL, very-low-density lipoprotein, TG, triglycerides. p values were determined by using Pearson’s r for normally distributed data and Spearman’s rho for skewed data. ∗p < 0.05 and ∗∗p < 0.01. Related to Figure S3.

To further interrogate the links among BAT, FFA, and TG kinetics, we performed post hoc univariate correlation and multivariable regression analyses. Plasma palmitate and FFA concentrations were negatively correlated with BAT volume (Figures 3A and 3B), and plasma palmitate clearance rate was positively correlated with BAT volume (Figure 3C). Plasma TG and VLDL-TG concentrations were also negatively correlated with BAT volume (Figures 3D and 3E), and the VLDL-TG clearance rate tended to positively correlate with BAT volume (p = 0.10) (Figure 3F), but the correlation was not statistically significant because of the greater variability in VLDL-TG clearance rate in the HBAT than the LBAT group and the small number of participants. We also found that different measures of BAT activity (total, mean, and max) were negatively correlated with plasma palmitate, FFA, VLDL-TG, and TG concentrations and positively correlated with palmitate and VLDL-TG clearance rates (Figure 3G). Age negatively correlated with VLDL-TG clearance rate but not with VLDL-TG and palmitate concentrations or palmitate clearance rate (Figure S3). To account for residual influences of potential confounders (i.e., age and body fat distribution) in the link between BAT and metabolic outcomes, we performed multivariable regression analyses with both age and body fat distribution as predictors (Table 2). BAT volume and BAT metabolic activity (defined as SUV_max_) were significantly associated with plasma palmitate clearance rate, independent of age. BAT volume and BAT metabolic activity were no longer significantly associated with VLDL-TG clearance rate when age was included as a predictor in the multivariate regression analysis.

**Table 2:** Multivariable regression estimates for the relationships among BAT, VLDL-TG, and palmitate clearance rates

Independent predictors	Beta coefficient	Standard error	Standardized beta	p value
**Dependent variable: Plasma palmitate clearance rate (L/h)**^a^				

Total BAT volume (mL)^a^	0.108	0.023	0.900	<0.001
Age (years)	0.002	0.002	0.282	0.18
SAT:VAT ratio	0.002	0.007	0.045	0.79

Total BAT SUV_LBM max_ (g/mL)^a^	23.7	5.5	0.890	<0.001
Age (years)	0.17	0.18	0.196	0.34
SAT:VAT ratio	−0.46	0.77	−0.113	0.56

**Dependent variable: VLDL-TG clearance rate (mL/h)**^a^				

Total BAT volume (g/mL)^a^	0.029	0.076	0.090	0.75
Age (years)	−0.010	0.006	0.415	0.16
SAT:VAT ratio	0.004	0.023	0.034	0.87

Total BAT SUV_LBM max_ (g/mL)^a^	1,542.1	897.4	0.384	0.10
Age (years)	−39.4	27.2	−0.314	0.16
SAT:VAT ratio	−97.4	124.1	−0.156	0.44

BAT, brown adipose tissue; SUV_LBM_, SUV normalized to lean body mass; VLDL, very-low-density lipoprotein; TGs, triglycerides.

a Values were log transformed for data analysis.

## Discussion

Increased plasma FFA and TG concentrations have been implicated in the pathogenesis of insulin resistance, type 2 diabetes, and coronary heart disease.^1^^,^^2^ We found that plasma FFA and TG concentrations were lower in women with overweight/obesity who have high BAT volume compared to women with overweight/obesity who have low BAT volume. These findings are consistent with most,^14^^,^^15^^,^^16^^,^^19^ but not all,^17^^,^^20^ studies that have previously investigated the relationship between BAT and plasma lipid concentrations. We evaluated the potential metabolic mechanisms responsible for differences in plasma lipid concentrations and found that FFA and VLDL-TG appearance rates in the circulation were not different between the HBAT and LBAT groups, but FFA and VLDL-TG plasma clearance rates (i.e., the volume of plasma cleared of palmitate or VLDL-TG per hour) were higher in the HBAT group than in the LBAT group. These data demonstrate that differences in basal plasma FFA and VLDL-TG concentrations during thermoneutrality in people with HBAT and LBAT are associated with more efficient clearance processes, rather than reduced production, in the HBAT compared to the LBAT group.

The concentrations of FFA and TG in plasma represent the balance between their release into the circulation and clearance from plasma by various tissues. We found that the rates of FFA and VLDL-TG release into the systemic circulation were not different between the HBAT and LBAT groups. However, FFA and VLDL-TG plasma clearance rates were higher in the HBAT than the LBAT group, which caused lower FFA and TG concentrations even though there were no differences in their appearance rates (or absolute disappearance rates during steady-state conditions). In post hoc correlation analyses, BAT volume and activity (total, mean, and max) correlated with palmitate clearance rate, and VLDL-TG clearance rate correlated or tended to correlate with BAT volume and activity (total and max). After adjusting the relationship between BAT and lipid kinetics for age and body fat distribution, plasma palmitate clearance rate was still significantly associated with BAT volume and activity, whereas VLDL-TG clearance rate was only marginally associated with BAT activity and was not associated with BAT volume. Taken together, our findings suggest that cold-activated BAT is an independent predictor of plasma FFA clearance rate but a weak predictor of VLDL-TG clearance rate.

Although it is possible that BAT per se causes higher lipid clearance in people with high BAT volume, the capacity to remove TG and FFAs from the circulation by BAT may be limited because of the small measurable amount of metabolically active BAT (assessed by using ^18^F-FDG-PET-CT), even in people with high BAT volume. By using microdialysis probes to assess glycerol release (an index of TG hydrolysis) from supraclavicular adipose tissue or [^18^F]-fluoro-6-thia-heptadecanoic acid (FTHA)-PET imaging to assess supraclavicular adipose tissue FFA uptake, it was estimated that the oxidation of intracellular TG in BAT was ∼1 μmol/100 g organ/min (∼1 g TG/day, representing ∼4% total body VLDL-TG production rate); FFA uptake was estimated to be 0.2–1.7 μmol/100 g organ/min (representing <1% FFA appearance rate in plasma) during thermoneutrality in people.^21^^,^^22^^,^^23^^,^^24^^,^^25^ In addition, even cold-activated BAT clears <1% ingested fatty acids (consumed as dietary TGs and delivered as chylomicrons into the systemic circulation).^26^ These findings suggest that BAT has minimal direct effects on total TG and FFA disposal during either thermoneutral or acute cold exposure conditions in people, which is distinctly different from the results obtained from studies conducted in rodents that found that BAT is responsible for about 10% total TG disposal during thermoneutrality^11^ and about 50% total TG disposal after prolonged cold exposure.^10^^,^^27^

Increased circulating levels of TG and FFA are part of the constellation of metabolic abnormalities associated with obesity.^28^ Efficient clearance of plasma TG and FFAs for storage in adipose tissue and oxidation in other tissues (e.g., skeletal and cardiac muscles, liver, BAT) helps maintain low plasma TG and FFA concentrations. The small amount of BAT in people, even in those with high BAT volume, make it unlikely that BAT per se is an important site for lipid disposal. Therefore, the increased clearance of TG and FFAs in people with high BAT volume must occur in other tissues than BAT. However, the tissue(s) responsible for this increased uptake is not clear. The mechanism responsible for the more rapid TG and FFA uptake by non-BAT tissues in people with high BAT volume is also not known, but it is possible that molecules (i.e., peptides, metabolites, microRNAs) secreted by BAT affect metabolic function in other tissues.^29^

In summary, women with overweight/obesity who have a large amount of BAT have lower plasma FFA and TG concentrations because of higher plasma VLDL-TG and FFA clearance rates than women with a low amount of BAT. The cellular mechanisms responsible for the increase in plasma lipid clearance are not known, but the small volume of BAT in people suggests that enhanced lipid metabolism in other organs is involved.

### Limitations of the study

Our study has several limitations. First, BAT volume declines with increasing age.^16^^,^^30^^,^^31^ Therefore, the HBAT group was younger than the LBAT group, which introduces the potential confounding effect of age on our study outcomes. We have previously found that age, per se, independent of alterations in body composition, plasma insulin concentrations, or insulin sensitivity, was not an important determinant of basal FFA or VLDL-TG kinetics.^6^^,^^32^ Nonetheless, we performed post hoc multivariable regression analyses to adjust the metabolic outcomes for age and found that age was not a significant predictor of plasma palmitate and VLDL-TG clearance rate. However, adjusting for age eliminated the statistical significance of the relationship between BAT and VLDL-TG clearance rate without affecting the association between BAT and palmitate clearance rate. Second, our study was conducted during thermoneutral conditions, when BAT is in a quiescent metabolic state, so we do not know if prolonged cold-induced BAT activation would have affected our study outcomes. However, data obtained during thermoneutrality are more clinically relevant than data obtained during cold exposure. Third, our study was conducted in women, so we cannot determine if these results also apply to men.

## STAR★Methods

### Key resources table

**Table:** 

REAGENT or RESOURCE	SOURCE	IDENTIFIER
**Critical commercial assays**		

Triglyceride assay	FUJIFILM Wako Pure Chemical Corporation	632–50991
Apolipoprotein B	Kamiya Biomedical Company	KAI-004

**Software and algorithms**		

MIM version 6.7	MIMSoftware	NA
SPSS version 29	IBM Corporation	NA
BioRender	BioRender	NA
Graphpad Prism version 10	Graph Pad Software	NA

**Other**		

[1,1,2,3,3-2H5]glycerol	Cambridge Isotope Laboratories	DLM-1229
[U-13C]potassium palmitate	Cambridge Isotope Laboratories	CLM-3943
[5,5,5-2H3]leucine	Cambridge Isotope Laboratories	DLM-1259

### Resource availability

#### Lead contact

Further information and requests for resources and reagents should be directed to and will be fulfilled by the Lead Contact, Maria Chondronikola (mc2425@medschl.cam.ac.uk)

#### Materials availability

This study did not generate new unique reagents.

#### Data and code availability

• All data reported in this paper will be shared by the lead contact upon reasonable request.
• This paper does not report additional code.
• Any additional information required to reanalyze the data reported in this work paper is available from the lead contact upon reasonable request.

### Experimental model and study particpant details

#### Participants

Twenty-five women with overweight or obesity (BMI 25.0–35.0 kg/m^2^) and high (≥20 mL) or no/minimal (<20 mL) total BAT volume in the neck and torso combined participated in this study (Table 1 and Figure S1), which was conducted at Washington University School of Medicine in St. Louis, MO. The study population was limited to women with overweight and obesity because VLDL-TG clearance is a more important determinant of plasma VLDL-TG concentration in women than men,^6^ and studies in rodents show BAT decreases plasma TG concentrations by increasing the clearance of TG-rich lipoproteins.^10^ The BAT volume threshold was determined based on previous investigations indicating that people with <20 mL of BAT demonstrated a minimal response to whole-body glucose disposal and FFA mobilization after cold-induced BAT activation.^33^^,^^34^ Out of the 29 women, 25 completed all study procedures related to the primary outcome of the study (VLDL-TG kinetics) (Table 1). Biopsy samples were collected from 10 of the 11 participants in the LBAT group and all the participants in the HBAT group. All participants completed a comprehensive screening evaluation that included a medical history and physical examination, an electrocardiogram, standard blood tests, and an oral glucose tolerance test. Potential participants were excluded if they had diabetes or other serious diseases, unstable body weight (>5% change during the last 2 months before entering the study), exercised regularly (≥30 min of intense exercise on more than 2 days/week or more than a total of 150 min of moderate or intense exercise per week), received medications (beta blockers, corticosteroids, etc.) that could affect the study outcomes or increase the risk of adverse effects (e.g., excessive bleeding) related to the study procedures, smoked cigarettes, consumed excessive amounts of alcohol (>14 units/week), were pregnant or lactating, received radiation exposure during the last year, or had metal implants that interfered with the imaging procedures.

#### Study approval

The study was approved by the Institutional Review Board of Washington University School of Medicine in St. Louis, MO. Written informed consent was obtained from all participants before their participation in this study (ClinicalTrials.gov NCT02786251).

### Method details

#### Study design

Each participant performed a BAT assessment study visit, which involved an intravenous [^18^F]FDG injection in conjunction with a PET-CT scan after about 6 h of mild cold exposure by using liquid conditioned garments, to determine BAT volume and BAT metabolic activity. About two days to two weeks later, participants completed two additional study visits: i) a metabolic study visit to assess plasma palmitate, VLDL-TG, and VLDL-apoB kinetics and to obtain subcutaneous abdominal adipose tissue biopsies and ii) a supraclavicular adipose tissue biopsy visit. Participants were asked to avoid exposure to high (>30°C) or low (<10°C) temperatures (e.g., sauna, winter sports and ice bath), and to maintain current weight and usual diet during their participation in the study. They were further instructed to refrain from excessive physical activity and consumption of alcohol, spices, and caffeine for at least three days before each study visit. Some participants opted not to complete all study procedures (Figure S1).

#### BAT assessment visit

Participants were admitted to the Clinical and Translational Research Unit (CTRU) the night before the study. At ∼ 0700 h, after participants fasted for 9-h overnight, a 6-h standard cooling protocol was performed to maximize non-shivering thermogenesis.^33^^,^^34^ During cold exposure, participants wore shorts, a sports bra, a T-shirt/hospital gown and a liquid-conditioned, whole-body cooling wrap (ThermoWrap, Belmont Medical Technologies, Billerica, MA). The use of cold-water perfused garments induces heat loss through both conduction and convection, which is more effective than cold ambient temperature alone (heat loss via convection only) in achieving maximal thermogenesis.^35^ The room and cooling equipment temperature were initially set to 22°C and decreased by 1°C in intervals of 60 min until shivering, determined by visual inspection of shivering or reported by the participants, was induced. Immediately upon shivering, cooling equipment temperature was increased by 1°C–2°C until shivering stopped. Validated questionnaires were used to assess the thermal comfort and sensation and self-reported shivering (ASHRAE thermal sensation scale).^36^^,^^37^^,^^38^ We used a personalized cooling protocol to ensure that the participants were comfortable during cold exposure and to minimize overt shivering. Core temperatures were measured using a telemetric pill (Core-Temp, HQ Inc., Palmeto, FL). Wireless probes (iButtons, Maxim, Dallas, TX) were used to measure the ambient temperature and participants’ skin temperature during the study visits as we have previously described.^39^ The average skin temperature was calculated as the average of the probe temperatures. Environmental and body temperatures along with self-reported shivering, thermal sensation and comfort were not different between the HBAT and LBAT groups (Table S2 and Figure S4).

After 5 h of exposure to cold, participants were transferred by wheelchair to the Center for Clinical Imaging Research (CCIR), which is a few minutes walking distance from the CTRU. Upon arrival in the CCIR, participants continued the cold exposure and 185 MBq of [^18^F]FDG was injected in an arm vein. Sixty minutes later, PET-CT (Siemens Biograph 40 PET/CT TruePoint/TrueView scanner; Siemens AG, Erlangen, Germany) imaging from the skull to the mid-thigh region was performed to evaluate BAT volume and BAT metabolic activity along with [^18^F]FDG uptake and radiodensity in other metabolically important tissues. The static PET images were reconstructed for each station utilizing the entire list-mode dataset as previously described.^40^ BAT volume and activity were quantified according to BARCIST 1.0.^41^ The analysis of PET-CT imaging was conducted by using MIM version 6.7 (MIM Software; Cleveland, OH). BAT volume was determined as the sum of BAT between the skull base and the knee by using a 3D-axial method.^42^ BAT was defined as a region with: i) radiodensity within the fat range (−190 to −10 Hounsfield units) and ii) [^18^F]FDG uptake above normal background, defined as a lean body mass-(LBM) adjusted standardized uptake value (SUV_LBM_) greater than 1.2 g/mL tissue/(lean body mass/total body mass).^41^ Maximal [^18^F]FDG uptake (SUV_max_) was defined as the voxel with the highest [^18^F]FDG uptake within all the segmented BAT depots. Average [^18^F]FDG uptake (SUV_mean_) was determined as the mean [^18^F]FDG uptake for all voxels within the BAT regions. BAT activity was calculated as the product of the total BAT volume and SUV_mean_. The radiodensity and metabolic activity (SUV_mean_) for other regions of interest (supraclavicular adipose tissue, liver, abdominal subcutaneous and visceral adipose tissue, and skeletal muscle) were determined as previously described.^43^

#### Metabolic study

To assess plasma palmitate, VLDL-TG, and VLDL-apoB kinetics, each participant completed a stable isotopically-labeled tracer infusion study (Figure S5) after they fasted for about 8 h overnight during thermoneutral conditions (26°C–28°C) while wearing shorts and a T-shirt or a hospital gown. Participants were admitted to the CTRU on the evening before the study. At 1900 h, they consumed a standard meal containing 30% of their estimated daily energy expenditure (by using the Mifflin-St Jeor equation with an activity factor of 1.25),^44^ 50% of total energy was provided as carbohydrates, 30% as fat, and 20% as protein. At 2200 h, they consumed a liquid snack (two cartons of Boost Original, Nestle USA, Bridgewater, NJ). Participants then fasted (except for water) and rested in bed until completion of the study the next day. At 0500 h the following morning, a catheter was inserted into a forearm vein to administer the stable isotopically-labeled tracers and a second catheter was inserted into a vein in the contralateral forearm to obtain blood samples. At ∼0600 h (*t* = - 4h), after a blood sample for the determination of background glycerol, palmitate, and leucine enrichment in plasma and VLDL-TG and VLDL-apoB were obtained, a constant infusion of [U-^13^C]palmitate (6 nmol/kg/min, dissolved in human albumin solution) was started and maintained until the end of the study to determine the palmitate appearance rate in plasma as an index of adipose tissue triglyceride lipolysis^4^ and the incorporation of plasma fatty acids into VLDL-TG.^6^ Four hours later, a bolus of [1,1,2,3,3-^2^H_5_]glycerol (75 μmol/kg, dissolved in 0.9% NaCl solution) was administered to determine VLDL-TG turnover,^6^^,^^45^ and a primed, constant infusion of [5,5,5-^2^H_3_]leucine (0.06 μmol/kg/min; priming dose, 4.2 μmol/kg; dissolved in 0.9% NaCl solution), was started and maintained for 12 h to determine VLDL-apoB turnover.^46^ Subcutaneous abdominal adipose tissue from the periumbilical area was obtained ∼2–3 h after the administration of the glycerol bolus.^47^ After administering lidocaine to numb the skin and underlying tissues, abdominal subcutaneous adipose tissue was aspirated through a 4-mm liposuction cannula (Tulip Medical Products, San Diego, CA) from the periumbilical area.

A subset of the participants (n = 9 in the LBAT group and n = 10 in the HBAT group) also completed an identical metabolic study visit during prolonged mild cold exposure. The cold exposure and thermoneutral metabolic study visits were performed in random order and approximately two weeks apart. For the cold exposure metabolic study, we implemented a 16-h cold exposure protocol that involved a 6-h cold exposure protocol as described in the BAT assessment visit followed by a 10-h exposure to ambient temperature of 19-20°C. We experienced technical issues and found high inter-subject variability that made the interpretation of the TG kinetic data unreliable and, thus, this manuscript exclusively focuses on the outcomes collected during the thermoneutral condition.

Blood samples were collected hourly between −4 h and 12 h; additional samples were collected at 5, 15, 30, 60, 90, and 120 min after the glycerol bolus injection to determine FFA, VLDL-TG, and VLDL-apoB concentrations, glycerol, and palmitate TTRs in plasma and VLDL-TG, and leucine TTRs in plasma and VLDL-apoB. Blood samples were collected in chilled tubes containing EDTA. Samples were placed on ice and plasma was separated by centrifugation within 30 min of collection. Aliquots of plasma were kept in the refrigerator for isolation of VLDL by ultracentrifugation immediately after the end of the study, as we have previously described.^48^ The remaining plasma samples were stored at −80°C. Plasma free glycerol, palmitate, and leucine tracer-to-tracee ratios (TTRs), the TTRs of glycerol and palmitate in VLDL-TG, and the TTR of VLDL-apoB were determined by electron-impact ionization gas chromatography-mass spectrometry (GC/MS, Agilent 5973 and 5977B systems, Santa Clara, CA).^45^^,^^49^ Plasma FFA concentrations were quantified by using gas chromatography (Agilent 5973 GC/MS, Santa Clara, CA) after adding heptadecanoic acid to plasma as an internal standard.^50^ Plasma total TG and VLDL-TG concentrations were determined by using a colorimetric enzymatic kit (FUJIFILM Wako Pure Chemical Corporation, Osaka, Japan).^51^ VLDL-apoB concentration was measured by using a turbidimetric immunoassay (Kamiya Biomedical Company, Seattle, WA).

#### Calculations of metabolic kinetics

The fractional catabolic rates (FCR, pools/h) of VLDL-TG and VLDL-apoB were determined by fitting the plasma free and VLDL-TG bound glycerol and plasma free and VLDL-apo bound leucine TTRs and concentrations to a compartmental model.^45^^,^^52^ The secretion rates of VLDL-TG and VLDL-apoB, which equal VLDL-TG and VLDL-apoB disposal rates during steady state conditions, were calculated as the product of the respective FCR, concentration, and plasma volume in liters,^52^ which was assumed to be 0.055 L/kg fat-free mass.^53^ VLDL-TG and VLDL-apoB plasma clearance rates, i.e., the volume of plasma cleared of VLDL-TG and VLDL-apoB per unit of time, were calculated as the product of FCR and plasma volume. The relative contribution of systemic and non-systemic fatty acids to VLDL-TG was calculated by the principle of isotopic dilution of the infused [U-^13^C]palmitate tracer by compartmental modeling analysis.^45^^,^^49^ Systemic fatty acids represent circulating FFA (mostly derived from adipose tissue TG lipolysis) that are taken up by the liver and directly incorporated into VLDL-TG or temporarily incorporated into rapidly turning over intrahepatic and intraperitoneal TG stores before incorporation into VLDL-TG; non-systemic fatty acids represent fatty acids that do not equilibrate with the infused [U-^13^C]palmitate tracer, including fatty acids released from preexisting, slowly turning over lipid stores in the liver and tissues draining directly into the portal vein, fatty acids resulting from lipolysis of plasma lipoproteins that are taken up by the liver, and fatty acids derived from hepatic *de novo* lipogenesis. Palmitate R_a_ in plasma was calculated by dividing the palmitate tracer infusion rate by the average plasma palmitate TTR value between 4 h and 6 h during physiologic and isotopic steady state.^34^ Plasma palmitate clearance rate was calculated by dividing palmitate R_a_ by the plasma palmitate concentration.^4^

#### Supraclavicular adipose tissue biopsy visit

After participants fasted for ∼10 h overnight at home, they were admitted to the CTRU in the morning. After remaining at thermoneutrality (∼26-28°C) for an additional 5 h, participants were transported by wheelchair to the CCIR where an interventional radiologist obtained a supraclavicular adipose tissue biopsy sample by using the PET-CT and CT-guided percutaneous needle biopsy technique we have previously described.^54^

#### Body composition

Body fat mass, fat-free mass, and subcutaneous abdominal and visceral adipose tissue masses were determined by using dual-energy X-ray absorptiometry (Lunar iDXA, GE Healthcare, Chicago, IL).

#### Diet assessment

Participants’ dietary intake was assessed by using a three-day diet record, which was analyzed using ASA24 dietary assessment tool (National Cancer Institute, Bethesda, MD). Seven participants from the LBAT group and ten participants in HBAT group provided complete records.

#### Adipose tissue processing, RNA isolation, cDNA synthesis, quantitative real-time PCR, and RNA sequencing

Tissue samples were rinsed with ice-cold saline immediately after collection, cleaned off connective tissue and blood and immediately frozen in liquid nitrogen. Approximately 100 mg of adipose tissue was used for extracting RNA by using the RNeasy Mini Kit (74104; Qiagen, Chatsworth, California) including an on-column DNAse digestion step. *UCP1* gene expression, relative to the housekeeping control gene ribosomal protein lateral stalk subunit P0 *(RPLP0)*, was determined by using an ABI 7500 Real-Time PCR System (Invitrogen, Carlsbad, California) and Fast SYBR Green Master Mix (4385618, Invitrogen) and the primers shown in Table S3.

### Quantification and statistical analysis

#### Statistical analysis

Results are presented as means ± SD for normally distributed data and as median and interquartile range for skewed data. Differences between groups were assessed by using Student’s t-test for normally distributed data and the Mann Mann–Whitney U test for skewed data. Within group comparisons of tissue-specific metabolic variables were assessed by using paired t-test for normally distributed data and the Wilcoxon rank-sum test for skewed data. Pearson’s r for normally distributed data and Spearman’s rho for skewed data were used to evaluate the correlation between the various variables of interest. In addition, multivariable regression analysis was performed to assess the relationships between BAT and VLDL-TG and palmitate kinetics and concentrations after adjusting for both age and body fat distribution. Statistical analyses were performed by using GraphPad v9 (GraphPad Software, San Diego, CA) and SPSS v27 (IBM, Armonk, New York). All statistical tests assumed a 95% level of confidence as significant.

#### Sample size estimation and statistical power

Based on our preliminary data (i.e., VLDL-TG clearance rate (mean and SD) 1146 ± 432 mL/h in the HBAT (n = 4) and 2016 ± 77.4 mL/h (n = 4) in the LBAT group), we estimated that 10 subjects in each group would be needed to detect the same difference in VLDL-TG clearance rates between HBAT and LBAT groups by using a two-sided t-test with a power of 0.8 and an α value of 0.05. These computations were performed by using G∗Power 3.1.9.4.^55^

### Additional resources

Clinical trial registration number NCT02786251, clinicaltrials.gov (https://clinicaltrials.gov/study/NCT02786251?tab=table).

## Supplemental information

Document S1. Figures S1–S5 and Tables S1–S3

Document S2. Article plus supplemental information
