# Supplementary material for: Very-low-density lipoprotein triglyceride and free fatty acid plasma kinetics in women with high or low brown adipose tissue volume and overweight/obesity
Source: Cell Rep Med. 2024 Jan 16;5(1):101370. doi: 10.1016/j.xcrm.2023.101370 (PMC10829791; doi:10.1016/j.xcrm.2023.101370)
Supplement: Document S1. Figures S1–S5 and Tables S1–S3 [file mmc1.pdf]

Cell Reports Medicine, Volume 5

Supplemental information

Very-low-density lipoprotein triglyceride and free fatty acid plasma kinetics in women with high or low brown adipose tissue volume and overweight/obesity

Maria Chondronikola, Jun Yoshino, Raja Ramaswamy, Joseph Daniel Giardina, Richard Laforest, Richard L. Wahl, Bruce W. Patterson, Bettina Mittendorfer, and Samuel Klein

SUPPLEMENTAL MATERIAL

Very low-density lipoprotein triglyceride and free fatty acid plasma kinetics in women with overweight/obesity and high or low brown adipose tissue volumes

Maria Chondronikola[1,2,3], Jun Yoshino[1], Raja Ramaswamy[4], Joseph Daniel Giardina[4], Richard Laforest[4], Richard L. Wahl[4], Bruce W. Patterson[1], Bettina Mittendorfer[1], Samuel Klein[1,5]

AFFILIATIONS:

[1] Center for Human Nutrition, Washington University School of Medicine, St. Louis, MO, USA

[2] Wellcome-MRC Institute of Metabolic Science-Metabolic Research Laboratories, Medical Research Council Metabolic Diseases Unit, University of Cambridge, Cambridge, UK

[3] Department of Nutritional Sciences and Dietetics, Harokopio University of Athens, Greece

[4] Department of Radiology, Washington University School of Medicine, St. Louis, MO, USA

[5] Sansum Diabetes Research Institute, Santa Barbara, CA, USA

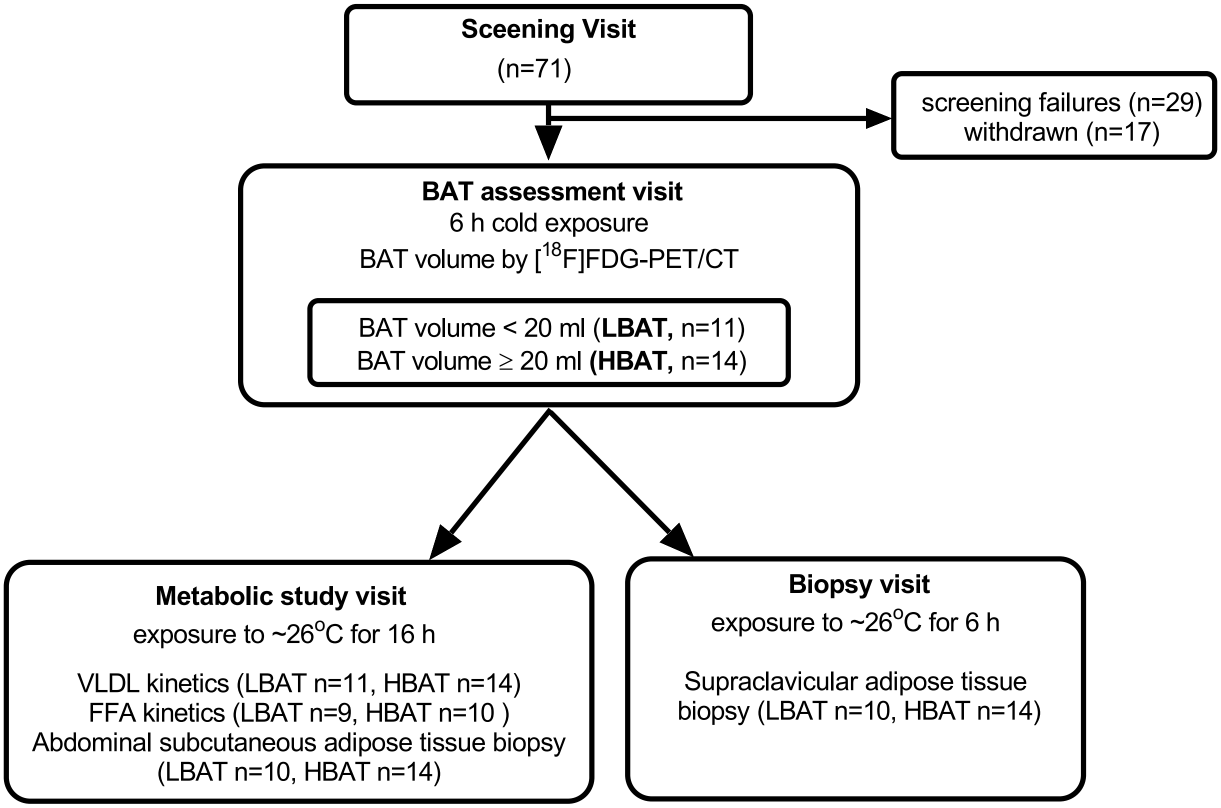

Figure S1. CONSORT diagram of the study. A smaller number of participants completed assessment of FFA kinetics because this assessment was added later in the study protocol. One participant in the LBAT group declined the abdominal and supraclavicular adipose tissue biopsy procedures. BAT: brown adipose tissue; FFA: free fatty acids; HBAT: high-BAT group; LBAT: low-BAT group; PET-CT: positron emission tomography - computed tomography; VLDL: very low-density lipoprotein; [^{18}F]FDG: 2-deoxy-2-[^{18}F]-fluoro-glucose. Related to **Figure 1**.

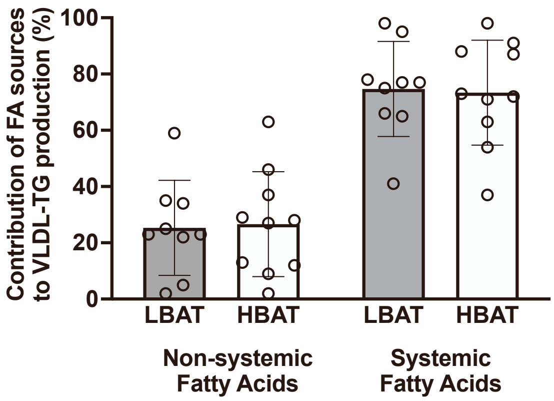

Figure S2. BAT and FA contribution to VLDL-TG production. Relative contribution of systemic FA (generated primarily by adipose tissue TG lipolysis) and non-systemic FA (generated primarily by lipolysis of intrahepatic TG) to VLDL-TG production in the LBAT (n = 9) and HBAT (n = 10) groups. BAT: brown adipose tissue; HBAT: high-BAT group, BAT volume ≥ 20 mL; LBAT: low-BAT group, BAT volume < 20 mL; FA: fatty acids; VLDL-TG: very low-density lipoprotein triglyceride. Data are means and SD. Related to **Figure 2**.

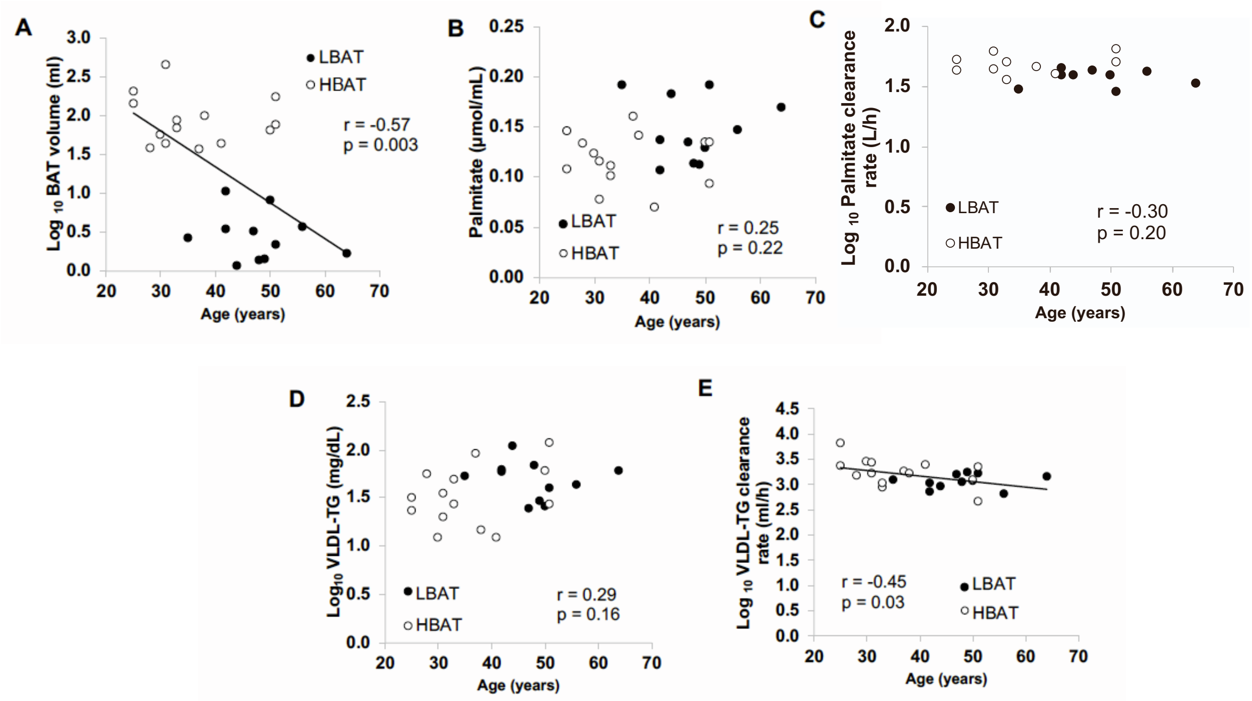

Figure S3. Relationships among age, BAT volume and lipid kinetics and concentrations. (A) Relationship between BAT volune and age. **(B)** Relationship between age and plasma palmitate concentration. **(C)** Relationship between age and plasma palmitate clearance rate. **(D)** Relationship between age and VLDL-TG concentration. **(E)** Relationship between age and VLDL-TG plasma clearance rate.BAT: brown adipose tissue; HBAT: high-BAT group, BAT volume ≥ 20 mL; LBAT: low-BAT group, BAT volume < 20 mL; VLDL-TG: very low-density lipoprotein triglyceride. Related to **Figure 3.**

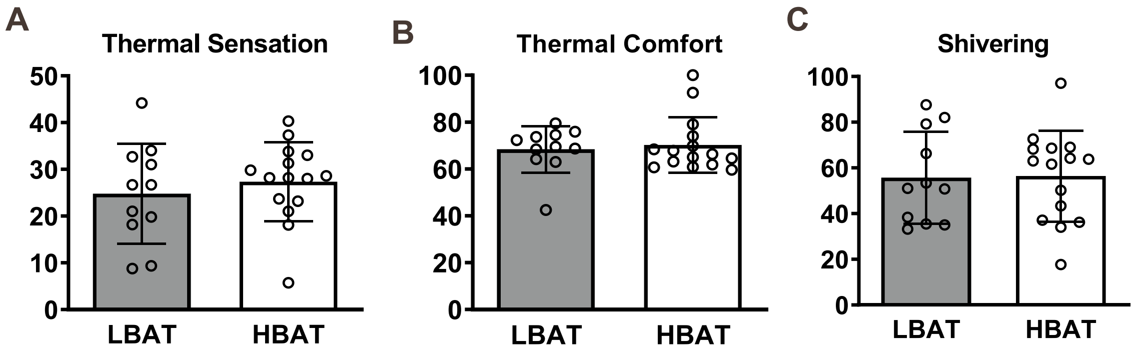

Figure S4. BAT status and sensory response to cold exposure in participants who completed the metabolic study visit. (A-C) Self-reported thermal sensation (0 = cold, 50 = neutral) **(A)**, comfort (0 = neutral, 100 = very uncomfortable) **(B)**, and shivering intensity (0 = Yes, 25 = sometimes, 75 = tense muscles, 100 = no) **(C)** in the LBAT (n = 11) and HBAT (n = 14) groups. Data are means and SD. BAT: brown adipose tissue; HBAT: high-BAT group, BAT volume ≥ 20 mL; LBAT: low-BAT group, BAT volume < 20 mL. Related to **Figure 1**.

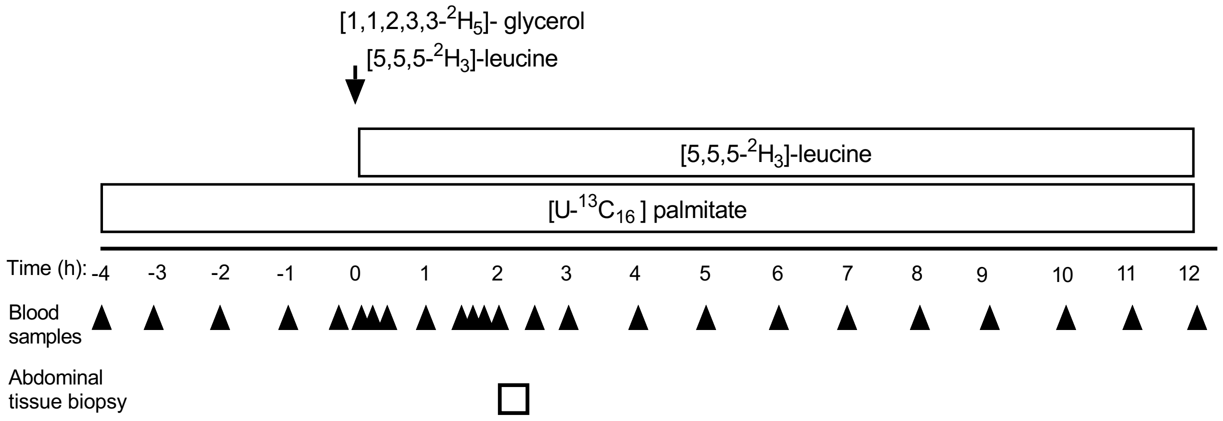

Figure S5. Metabolic study protocol. The metabolic study included intravenous infusions of stable isotopically labeled tracers to assess free fatty acid and very low-density lipoprotein apolipoprotein B and triglyceride kinetics. Blood samples were collected hourly between -4 h and 12 h; additional samples were collected at 5, 15, 30, 60, 90, and 120 min after the glycerol bolus injection at t = 0 h. Abdominal subcutaneous adipose tissue biopsy samples were collected at time t = 2-3h. Related to **Figure 2**.

	LBAT (n = 7[a])	HBAT (n = 10[a])
**Energy intake (kcal/d)**	1812 (1321, 1978)	1629 (1219, 2080)
**Energy intake (kcal/kg FFM/d)**	33.6 (27.4, 42.4)	33.2 (25.7, 40.0)
**Protein intake (g/d)**	85 ± 27	69 ± 22
**Fat intake (g/d)**	74 (72, 92)	61 (53, 95)
**Carbohydrate intake (g/d)**	168 (129, 203)	182 (134, 199)
**Protein intake (% of energy intake)**	19 ± 5	17 ± 6
**Fat intake (% of energy intake)**	40 (38, 44)	38 (37, 42)
**Carbohydrate intake (% of energy intake)**	38 (37, 42)	45 (42, 48)

Table S1. Participants' dietary intake. [a] Four participants in the LBAT group and four participants in the HBAT group did not complete diet records. Data are mean ± SD or median (quartiles) for skewed data. BAT: brown adipose tissue; HBAT: high-; LBAT: low-BAT group, BAT volume < 20 mL. Related to **Figure 1**.

	LBAT (n = 11)	HBAT (n = 14)
**Cooling wrap temperature (º C)**	22.9 ± 1.4	22.1 ± 0.9
**Room temperature (ºC)**	21.4 ± 0.7	21.5 ± 1.4
**Core temperature (ºC)**	37.0 (36.8, 37.3)	37.2 (37.1, 37.3)
**Heat production (water temperature in-out, ºC)**	0.8 ± 0.3	0.9 ± 0.3
**Mean skin temperature (ºC)**	30.0 (29.5, 30.7)	29.3 (28.8, 29.8)

Table S2. Environmental and body temperatures during cold exposure in participants who completed the metabolic study visit. A personalized cooling protocol was implemented to minimize overt shivering. Data are mean ± SD for normally distributed data or median (interquartile range) for skewed data. BAT: brown adipose tissue; HBAT: high-BAT group, BAT volume ≥ 20 mL; LBAT: low-BAT group, BAT volume < 20 mL. Related to **Figure 1**.

Gene	Accession No.	Forward (F) and Reverse (R) Primer
UCP1	NM_021833	F: AGGTCCAAGGTGAATGCCC<br>R: TTACCACAGCGGTGATTGTTC
RPLP0	NM_001002	F: GTGATGTGCAGCTGATCAAGACT<br>R: GATGACCAGCCCAAAGGAGA

Table S3. Sequence of primers used for reverse transcription polymerase chain reaction. Related to **Figure 1**.
